# Immune Resistance and Anti-Tumor Immunity in the Ovarian Tumor Microenvironment

**DOI:** 10.3390/cells15212004

**Published:** 2026-11-03

**Authors:** Patrick J. Stiff, Cheryl M. Czerlanis, Ronald K. Potkul, William Small, Maureen L. Drakes

**Affiliations:** 1 Department of Medicine, Cardinal Bernardin Cancer Center, Stritch School of Medicine, Loyola University Chicago, Building 112, 2160 South First Avenue, Maywood, IL 60153, USA; 2 Division of Hematology, Oncology and Palliative Care, UW School of Medicine and Public Health, University of Wisconsin (UW) Carbone Cancer Center, 600 N. Highland Avenue, K4/530, Madison, WI 53792, USA; cczerlanis@medicine.wisc.edu; 3 Department of Obstetrics and Gynecology, Cardinal Bernardin Cancer Center, Loyola University Chicago, 2160 South First Avenue, Maywood, IL 60153, USA; 4 Department of Radiation Oncology, Cardinal Bernardin Cancer Center, Loyola University Chicago, 2160 South First Avenue, Maywood, IL 60153, USA; wmsmall@lumc.edu

**Keywords:** ovarian cancer, tumor microenvironment, T regulatory cells, macrophages, dendritic cells, neutrophils, clinical trials, novel therapies

## Abstract

**Highlights:**

**What are the main findings?**

- The ovarian tumor microenvironment (TME) consists of heterogenous dysfunctional cell subsets that interact with cancer cells through direct contact or by the elaboration of soluble molecules and confer rigid and complex immune suppression.
- In high-grade serous ovarian cancer (HGSOC), the cellular and structural architecture of the tumor stroma actively facilitates cancer initiation, progression, metastasis, and/or chemoresistance.

**What are the implications of the main findings?**

- The optimum therapy efficacy for HGSOC will likely be achieved with the use of combined therapies designed to simultaneously target multiple barriers to anti-tumor responses in the TME.
- Novel tools such as spatial imaging, spatial transcriptomics, and spatial proteomics are useful for profiling the HGSOC TME to reveal unique biomarkers in patients that will enable the design of effective precision medicine.

**Abstract:**

Ovarian cancer is a lethal gynecologic malignancy with limited effective treatment options. It is expected that in 2026 there will be about 21,010 new cases of ovarian cancer diagnosed and about 12,450 deaths within the United States. Unfortunately, conventional therapies such as chemotherapy and radiation have limited efficacy for high-grade serous ovarian cancer (HGSOC) and also produce significant toxicity. HGSOC is characterized by an ovarian tumor microenvironment (TME) consisting of immune inhibitory T regulatory cells and inhibitory tumor-associated macrophages, including myeloid-derived suppressor cells (MDSCs) and M2 cells, which confer immune suppression. Immunocompetent T cells, tumor-suppressing M1 cells, natural killer cells, and other cellular subsets are also present. The TME is further shaped by soluble molecules, extracellular matrix, and other structural components, as well as the presence of blood vessels and ascitic fluid in the peritoneal cavity, which aid in the distribution of cancer cells to the omentum where more lesions soon develop. This review outlines the heterogenous immune cell subsets in the ovarian TME and other structural targets that promote or reduce cancer burden. Most single-agent immune strategies have shown limited efficacy in unselected HGSOC populations due to the complex immune-suppressive nature of the TME. Here, we also discuss novel combination treatments in clinical trials or in clinical practice for HGSOC. Advances in understanding the ovarian TME will reveal new immunotherapy and drug targets for translational medicine and aid in determining effective drug and immunotherapy combinations, which may ultimately lead to improved overall survival in HGSOC.

## 1. Introduction

Ovarian cancer is the leading cause of death from gynecologic cancers [1]. High-grade serous ovarian cancer (HGSOC) is discovered at advanced stages because of its non-specific symptoms and lack of effective screening tools. Platinum-based chemotherapy is initially effective for HGSOC patients, but these patients soon become resistant to these agents leading to disease recurrence and death [2,3,4,5].

In the quest to improve survival for this deadly cancer, newer treatments include immune checkpoint inhibitors (ICIs) and other immunotherapies, such as dendritic cell (DC) vaccine therapies, poly(ADP-ribose) polymerization (PARP) inhibitors, targeted therapies, and adoptive T-cell and natural killer (NK) cell therapies [6,7,8,9,10,11]. These and other strategies are being developed to target cancer cells, immune cells, or other suppressive components of the ovarian tumor microenvironment (TME). To date, most of these therapies have not reached the level of success needed to significantly impact HGSOC overall survival (O/S). However, one recently FDA-approved agent is Mirvetuximab Soravtansine (Elahere), a first-in-class antibody-drug conjugate (ADC) that targets folate receptor alpha (FRα) expressed on tumor cells. This agent that targets tumor cells in the TME is approved for HGSOC patients whose tumor cells have high FRα expression [12]. Another recently FDA-approved regimen is the use of pembrolizumab (Keytruda) (targeting immune checkpoints (ICs) in the TME) plus chemotherapy, an immunotherapy regimen paired with weekly paclitaxel (with or without bevacizumab) approved for patients with platinum-resistant disease, with PD-L1-expressing tumors [13]. A better understanding of the complexity of the ovarian TME will provide insight into designing best targeting strategies and the development of more effective treatment options.

Ovarian cancer is characterized by histological subtypes. Approximately ninety percent of ovarian cancer is of epithelial origin [14,15], including ovarian, fallopian tubes, and other sites in the pelvis. The other ten percent that are of non-epithelial origin are generally less aggressive than epithelial ovarian cancer. Epithelial ovarian cancer comprises mainly four groups: serous, endometroid, clear cell, and mucinous. Serous ovarian cancer is categorized as low grade (5 percent), mixed histology, or high grade (70 percent) (summarized in [15]). HGSOC is notorious for its late discovery and for developing early resistance to first-line therapies such as cytoreductive surgery and platinum-based chemotherapies [2,3,4,5].

The HGSOC TME consists of T regulatory cells, immunocompetent T cells, a myriad of macrophage subsets, NK cells, soluble factors, cancer cells, extracellular matrix components, vasculature, and other components coalescing into a series of networks, which favor immune suppression and cancer progression [8,15,16,17]. This review is a comprehensive summary of the role of the aforementioned TME elements as well as cells found in lower numbers in the HGSOC TME, such as gamma delta T cells, invariant NK T cells, and innate lymphoid cells. We discuss the potential of these TME components to hinder or advance HGSOC progression. We also discuss novel treatments for HGSOC, which are designed to overcome TME-suppressive mechanisms, as well as combinations of treatments that may be more effective than single therapy in improving disease prognosis and O/S. We conclude with a discussion of novel tools to study biomarkers in the HGSOC TME and how new drug strategies may be more precisely administered for the best outcome in precision medicine for this disease.

## 2. Immune Cells Regulate the Tumor Microenvironment

### 2.1. T Cells Tune the Ovarian Tumor Microenvironment

The increased presence of immune-competent tumor-infiltrating lymphocytes (TILs) in the HGSOC TME is reported by many to favor survival whereas T-cell exclusion is associated with worse prognosis [18,19,20]. In a study of 5500 patients of which 3196 were classified as HGSOC, TILs were associated with better outcomes regardless of the extent of surgical cytoreduction or chemotherapy regimens [21]. Tumors have previously been classified in a three-tier system based on the abundance and location of cytotoxic T cells. T-cell-inflamed tumors (also called hot tumors) are characterized by T-cell enrichment in the tumor deposits (islets) and in tumor-associated stroma, as well as increased PD-L1 expression. These hot tumors are more responsive to ICIs [22,23,24]. T-cell-excluded tumors (called altered tumors) are those in which CD8+ T cells are confined to the periphery and not infiltrated in the tumor mass. Non-inflamed tumors (called immune desert tumors or cold tumors) are the most T-cell-deficient tumors, both within the tumor as well as surrounding the tumor. Cold tumors are generally marked by the presence of immune-suppressive cells, including TAMs, MDSCs, and T regs [22,23,24].

A more standardized classification system for immune cells infiltrating ovarian cancer tumors was recently established by the use of immunohistochemistry (IHC) staining and evaluation, multiplex immunofluorescence (mIF) staining, and digital pathology analysis [25]. Five independent study cohorts were used for several cell subset studies. These cohorts consisted of a total of 697 tumor samples, of which 566 were of primary tumor origin and 131 from recurrent tumors [25].

These authors used an algorithm to capture the heterogeneity of CD8+ T-cell densities and spatial distribution in whole tissue slides. They converted the mean of five CD8+ T cells per high-power field (hpf) by IHC to a mean of 21 CD8+ T cells/mm^2^ on whole formalin-fixed paraffin-embedded (FFPE) slides stained by IF. Firstly, they tested the strength of this T-cell density model in primary tumors from two of the five clinical cohorts (IMCOL and UPENN cohorts) under study. It was found that tumors infiltrated by a mean of greater than 21 CD8+ T cells/mm^2^ had significantly longer survival than those with <21 CD8+ T cells/mm^2^ even after adjustments were made for optimal residual disease at the time of first surgery. These investigators identified four different immune phenotypes in the treatment of naïve ovarian cancer: purely inflamed, mixed inflamed, excluded, and desert tumors [25]. In this method, they developed a two-step algorithm to define a CD8+ T-cell-based immune classifier considering both cell density across whole tissue and heterogeneous CD8+ T-cell distribution within tumor/stroma compartments of each region of interest (ROI). The fraction of inflamed subregions (ROIs with >21 CD8 + T cells/mm^2^) in the tumor was categorized as purely inflamed if the percentage of inflamed ROIs was >70%. Tumor tissues with a percentage of inflamed ROIs between 50% and 70% were placed in the mixed inflamed group. When less than 50% of ROIs were inflamed in the tumor, they considered the stroma. The sample was placed in the excluded category if >10% of ROIs showed >21 CD8+ T cells/mm^2^ in the stroma. Finally, if <10% of ROIs showed >21 CD8+ T cells/mm^2^ in the stroma, the sample was placed in the desert category [25].

When the aforementioned CD8+ T-cell threshold was further applied to a third cohort (HiTide-UPENN), it was consistently found that long-term survivors were those with purely and mixed inflamed tumors when compared to those with excluded and desert tumors. They next used tissue microarrays (TMAs) from two large patient cohorts to confirm their findings. These cohorts were from the Nurses Health Study (NHS) and the NHSII and consisted of 418 patients. Even after several patient parameters were taken into account (such as tumor subtype, stage, and age), patients with mixed inflamed, excluded, and desert tumors had greater hazard ratios for death than patients with purely inflamed tumors [25]. Consistent with other studies, these authors also confirmed that samples with higher densities of CD3 T cells in both intratumoral and stromal locations were more beneficial to survival [18,19,20,21,26]. In a meta-analysis of 19 studies and 6004 patients CD3+, CD4+, CD8+, and CD103+ TILs were associated with longer O/S and progression free survival (P/FS) [27]. 

CD4+CD25+FoxP3 T regulatory cells (Tregs) in ovarian cancer have been the focus of several studies. They have been reported as a negative prognostic indicator by many investigators [28]. These cells may release pro-tumor cytokines, including interleukin-10 (IL-10) and transforming growth factor beta (TGF-β) by cell–cell contact mechanisms. It was found that when FoxP3+ cells were located within lymphoid aggregates surrounding tumors, these cells were significantly associated with reduced patient survival (*p* = 0.007) [29]. In mice, FoxP3+ T regs activated the Sirt1- ERK1/2- STAT-3 signaling pathway and suppressed NFK-β activity as a mechanism of promoting M2 macrophage polarization [30].

The classification of T regs encompasses many T-cell subsets, which suppress the responses of immune cells [31]. For example, whereas CD8+ T cells primarily kill tumor cells by recognizing tumor-specific peptides on MHC class I molecules, then releasing perforin and granzyme B or TNF-α to trigger death [32], in the immunosuppressive ovarian TME these CD8+ T cells may become dysfunctional and exhausted and upregulate inhibitory receptors such as the immune checkpoint (IC) receptor PD-1 [33,34,35]. T cells expressing high levels of IC are usually classified as negative immune regulators. ICs are generally but not exclusively expressed on T cells. Each IC usually has multiple ligands or binding sites on antigen-presenting, tumor, or other cells. Binding of ICs to ligand(s) usually results in reduced T-cell activity or lowered immune responses [33,34,35].

The most studied IC is programmed death-1 (PD-1; CD279). When PD-1 on T cells engages with programmed death ligand-1 (PD-L1; CD274) on macrophages or on tumor cells, this inhibits T-cell activity and leads to T-cell exhaustion. Antibodies blocking PD-1/PD-L1 linkage are FDA approved and show good efficacy in several solid cancers, such as melanoma, lung cancer, and renal cancer [36,37,38,39,40], but to date only show limited benefits in HGSOC [41,42,43,44].

Another IC, cytotoxic T-lymphocyte-associated protein-4 (CTLA-4), binds to receptor B7 on myeloid cells with higher affinity than CD28 binding to B7 (the latter gives a good anti-tumor response). The binding of CTLA-4 to B7 suppresses early T-cell activation leading to T-cell tolerance [36]. Other ICs in the ovarian TME include lymphocyte-activation gene-3 (LAG-3; CD223), which binds to MHC-II on antigen-presenting cells (APC) with higher affinity than CD4 binding to MHC-II, galectin-3, and other ligands. IC T-cell immunoreceptor with immunoglobulin and immunoreceptor tyrosine-based inhibition motif domain (TIGIT) binds to CD155 (PVR) on DCs and macrophages. T-cell immunoglobulin and mucin domain-containing protein 3 (TIM-3; CD366) binds to galectin-9. The presence of more than one IC on TILs may indicate an intense state of immune exhaustion and the use of combination ICI regimens may be a more effective therapeutic strategy in such cases [33,34,35,45].

The story of IC ligation activity in cancer is complex, such that there may be synergy between different IC pathways as well as with other immunosuppressive phenomena, such as tumor-associated macrophages (TAMs) and myeloid-derived suppressor cell (MDSC) aggregation. It is therefore likely that strategies designed to target IC axis activity in ovarian cancer will be more effective at reversing T-cell exhaustion if administered with other therapies.

### 2.2. Gamma Delta T Cells

The αβ T cell, the predominant T-cell population in the host, are critical for MHC-restricted antigen recognition. Another subset, gamma delta (γδ) T cells, makes up 1–10% of circulating T cells, but is found in higher percentages in tissues including the gut epithelium, spleen, liver, and other sites. In the normal host, γδ T cells are known to colonize the normal mucosa, where they protect against pathogens. These cells usually have different phenotypes and functions to their circulating counterparts and are crucial for local immune surveillance [46,47].

γδ T cells can be activated through the γδ receptor (T-cell receptor; TCR). On activation, they release perforin, granzymes, and IFN-γ, or kill through cell death ligands, like Fas and TNF-related apoptosis-inducing ligand (TRAIL)-R. These cells recognize antigens in an MHC-independent manner and, as such, respond more rapidly to stressed or transformed cells like in the TME, where they can recognize and attack malignant cells without the requirement for antigen processing and presentation [47]. The γδ T-cell subset can exert direct cytotoxicity against cancer cells and also boost anti-tumor effects through the release of cytokines, which influence other cells [46,48]. Human γδ T cells are also activated by NK cell receptors, a process not requiring TCR signaling. Activation through receptors NKp30, NKp44, and particularly NKG2D stimulates the cytotoxic activity of γδ T cells [49]. γδ T cells are also activated by butyrophilins and similar family molecules such as BTNL2 and BTN2A1, which is expressed in the uterus and fallopian tubes [46,50,51]. There is not yet a clear picture of the role of ovarian cancer-derived γδ T cells in HGSOC, but some investigators found that these cells were able to suppress the growth of ovarian cancer cells [52,53].

Over the past decade, several clinical trials on lung cancer, renal cancer, and multiple myeloma amongst other diseases have evaluated γδ-based therapies [46,48]. More evaluations need to be done on these cells in cancer, but it is anticipated that modifications of this cell subset may be a promising avenue for effective immunotherapy.

### 2.3. Invariant NKT Cells

NKT cells are a heterogenous group of cells that differ in phenotype and function [54,55]. The most studied group of these cells, invariant NKT (iNKT) cells, make up about 0.01%- 1% of T cells, and consists of several subgroups based on the cytokine profiles they secrete [55,56]. They are innate-like, non-conventional T cells that express TCR, NK receptors, and cytokine receptors, and are important in cancer surveillance [55,57]. Human iNKT cells express an invariant T-cell receptor, Vα24Jα18, as well as CD1d, a molecule related to MHC class-I, which presents self-lipids, glycolipids, and microbial antigens [55,58].

Activated iNKT cells can eliminate cancer cells in a CD1d-dependent manner. These cells recognize glycolipid antigens presented by CD1d molecules on tumor cells leading to direct cytotoxic responses [59]. They can kill tumor cells and can also regulate the TME to boost immune responses [58,60]. When activated, iNKT cells release great quantities of cytokines (IFN-γ, IL-12, IL-18, and IL-4 among others), which co-ordinate the activities of DCs, NK cells, and cytotoxic T cells. These cells can deplete TAMs and MDSCs and recruit effector cells to improve their function. They promote DC activation and maturation leading to more efficient T-cell stimulation [55,56,60,61]. Based on the aforementioned activities of these cells, they can potentially target ovarian cancer and the TME through several pathways, and can provide rapid multifaceted anti-tumor responses involving several immune cells. These potent cytotoxic cells are rare in vivo, but can be prepared on a larger scale under sterile in vitro conditions and are indeed promising as potent anti-cancer agents [62,63].

### 2.4. Opposing Roles of Macrophages in the Ovarian TME

Tumor-associated macrophages (TAMs) are the largest group of immune cells in the HGSOC TME [64,65]. One of the notable functions of these cells is that they can enhance ovarian cancer cell migration by secreting soluble molecules, such as TGF-β [66]. They are generally recruited from blood monocytes or sometimes originate from resident peritoneal macrophages. Both of these TAM groups exhibit phenotypes such as CD163 and CD206, as well as similar levels of genes for antigen-presenting cells and phagocytosis [17,67,68,69]. Importantly, TAMs in the TME exhibit higher levels of genes associated with extracellular matrix (ECM) remodeling [70]. TAMs mediate epithelial–mesenchymal transition (EMT), which promotes tumor progression. In this process, there is a downregulation of epithelial markers such as E-cadherin and upregulation of vimentin, snail, fibronectin, and alpha-smooth muscle actin. In EMT, epithelial cells change their phenotype to motile mesenchymal cells and aggressively migrate and invade tumors [71,72]. The process of EMT is fostered by several TAM products, including TGF-β, hepatocyte growth factor (HGF), and epidermal growth factor (EGF) [73].

It is reported that CD68+ and CD206+ TAMs are higher in patients with stage III/IV ovarian cancer than in patients with stage I/II diseases or patients with benign ovarian disease. These TAM groups (enriched for CD68 and CD206) were also higher in patients with lymphatic invasion than in those without lymphatic involvement [74]. The plasticity of TAMs is well-documented. For example, TAMs are high secretors of CCL18, a chemokine that promotes tumor cell migration and metastasis [75]. This chemokine can be blocked with interferon gamma (IFN-γ) secreted by CD4+ and CD8+ T cells, causing TAMs to switch to an anti-tumor phenotype [76].

IFN-γ in the TME promotes the emergence of M1 immunocompetent, classically activated macrophages, which are effective against microorganisms and cancer. M1 macrophages are secretors of IL-12, IL-23, and TNF-α, and are associated with improved survival in HGSOC patients [77]. M2 macrophages are a pro-cancer phenotype associated with poor outcomes in cancer patients [78]. A meta-analysis of 794 ovarian cancer patients evaluating 9 studies showed that a higher tumor M1/M2 ratio was indicative of improved ovarian cancer prognosis [79]. One ovarian cancer study found that suppressing T regs and blocking PD-1/PD-L1 linkage promoted the conversion of T regs to T helper cells, increased the ratio of M1 to M2 TAMs, and prolonged survival in mice [80].

In 246 primary ovarian tumors studied, inflamed tumors had the highest proportions of activated TAMs or myeloid cells expressing higher CD86 (co-stimulatory molecule) and pSTAT-1, suggesting the potential of greater anti-tumor immune responses in inflamed tumors [25]. When using RNA sequencing in 200 treatment-naïve ovarian cancer patient samples, it was found that in inflamed phenotype tumors (the highest T-cell index in tumors and stromas), there were highest levels of myeloid cell-related antigen presentation signatures, DC maturation signatures, and M1 macrophage signatures [25]. Signatures related to cancer progression and therapy resistance, such as EMT, ECM remodeling, and angiogenesis, were more prevalent in excluded and desert tumors where there were worse survival outcomes [25].

MDSCs (myeloid MDSCs) are a notorious type of TAMs known for HGSOC progression, increased tumor burden, metastasis, and resistance to immunotherapy [81,82,83]. In the ovarian cancer TME, there is a correlation between elevated levels of MDSCs and reduced survival. MDSCs migrate to the ovarian TME in the presence of CXCR4. Reports indicate that binding of the CXCR4 receptor to chemokine CXCL12 secreted under the influence of prostaglandin E2 (PGE2) is associated with HGSOC progression [84,85,86]. MDSCs also secrete several other molecules that abrogate cytotoxic T-cell responses. These include cyclooxygenase-2 (COX-2), an essential enzyme for PGE-2 synthesis, which facilitates the differentiation of DCs (CD1a+ cells) to MDSCs (CD14+CD33+CD34+ cells) [87].

**Figure 1:**
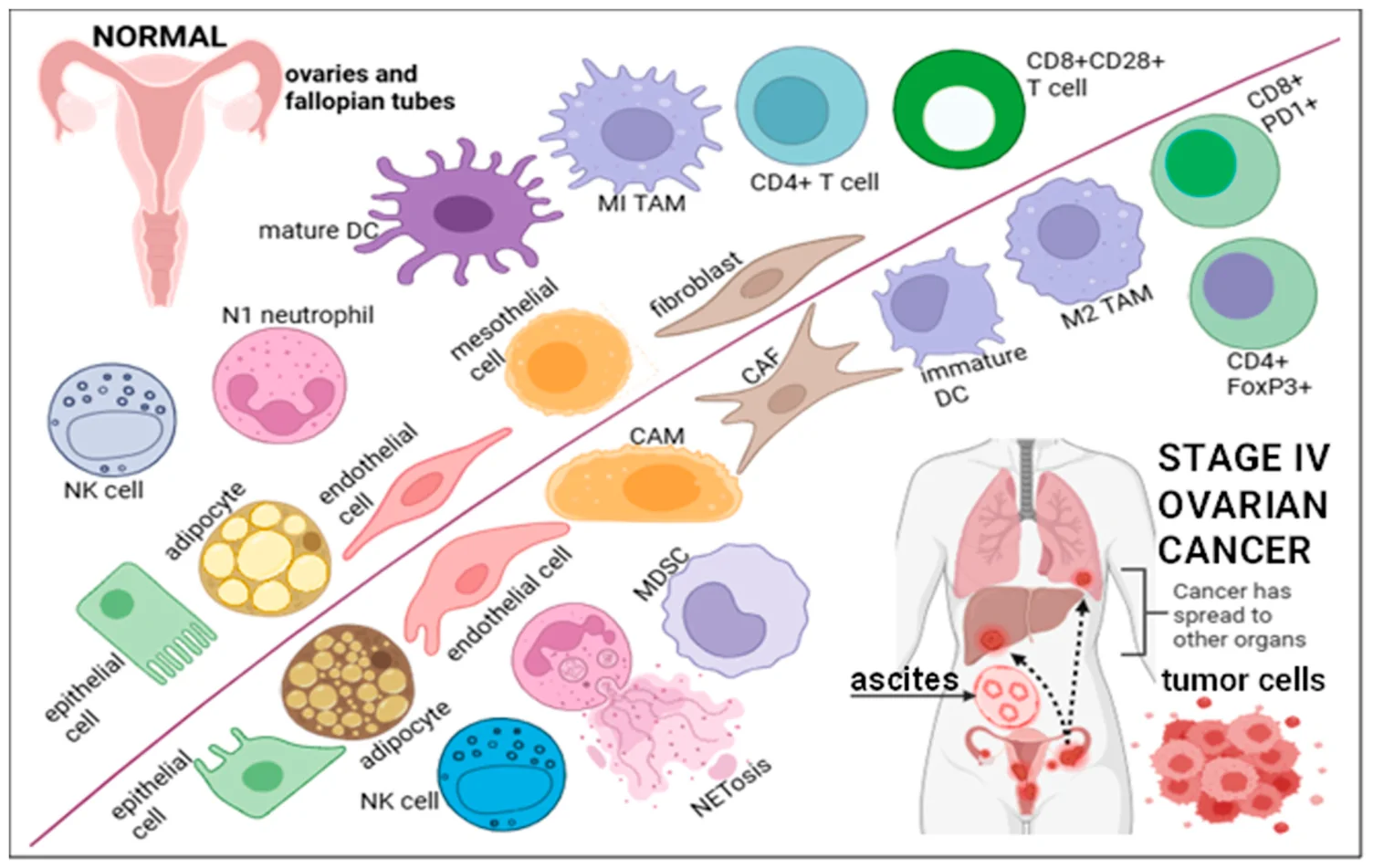
Cell subsets which favor a healthy reproductive system versus ovarian cancer. The upper panel represents cells that maintain a normal-functioning immune state in individuals. CD8+ T cells of different phenotypes and CD4+ T cells elaborate Th1 cytokines (IFN-γ, TNF-α, and IL-2) and contribute to normal homeostasis and immunocompetence. Similarly, M1 TAMs enhance anti-tumor responses and mature DCs are efficient at recognizing foreign or altered cells and antigen processing and presentation to effector T cells, which elicit cytotoxic responses. N1 TANs aid in the recruitment of CD8+ effector T cells. NK cells are important for killing foreign or altered cells through several cytotoxic mechanisms. Endothelial cells line and maintain the integrity of blood vessels, whereas epithelial and mesothelial cells are crucial for defense and structural integrity in the abdominal cavity and other sites. Adipocytes are important for lipid storage and endocrine signaling, whilst fibroblasts respond to tissue injury. The lower panel shows altered and dysfunctional cell types, which foster progression and metastasis in HGSOC patients. Under the influence of soluble molecules elicited in cancer, cells become altered in morphology, phenotype, and function. CD8+PD1+ and CD4+FoxP3+ exhausted/regulatory immunosuppressive cells recruit other negative regulators such as M2 TAMs and MDSCs, which promote HGSOC progression, increased tumor burden, and resistance to therapy. Dysfunctional NK cells can no longer perform their cytotoxic functions to eliminate tumor cells. Neutrophil (N2) NET formation may occur through NETosis, releasing IL-10 and attracting T regs fostering immune suppression and limiting T-cell proliferation. CAFs remodel the ECM and release cytokines, which stimulate metastasis of tumor cells. Endothelial cells promote angiogenesis and mesothelial cells establish niches of tumor cells for metastasis. Epithelial cells change form and initiate the process of EMT, which is essential for cancer progression. Figure 1 was created with Biorender.com.

Other negative immune regulators secreted by MDSCs include indoleamine 2,3-dioxygenase (IDO)**,** arginase-1 (ARG-1), IL-10, nitric oxide synthase-2 (NOS-2), and vascular endothelial growth factor (VEGF) [17,88]. Figure 1 is a schematic representation of cell subsets that are associated with healthy individuals compared with those that favor cancer initiation and progression.

### 2.5. Control of the Ovarian TME by Dendritic Cells

In HGSOC and other cancer TMEs, DCs are few in number and mostly have an immature phenotype with a characteristic low expression of co-stimulatory molecules, and as such are capable of mounting only minimal immune responses [89]. DCs may present exogenously captured peptides to CD4 T cells through the MHC class II complex and endogenous peptide antigens to CD8 T cells through the MHC class I complex. DCs also present exogenously captured antigens as MHC class I-associated peptides (cross-presentation) [90,91,92,93].

For efficient antigen presentation, DCs must have three signals: a cognate antigen, co-stimulatory molecules on the DC cell surface (CD40, CD80/B7-1, and CD86/B7-2, the latter two that bind to CD28 on T cells), and thirdly, the presence of pro-inflammatory cytokines (IFN-γ and IL-12). If these three stages of activation can occur in sequence, T cells are driven to migrate, proliferate, and attack malignant cells [94,95].

Briefly, immature DCs express CXCR3, CXCR4, CCR1, CCR2, CCR5, and CCR6. The presence of antigen causes DCs to mature, upregulate co-stimulatory molecules, downregulate chemokine receptors associated with immature DCs, and upregulate CCR7, which guides DCs to lymph nodes to present antigens to immune cells (T cells). These mature DCs bearing high expression of CD40 crosslink with CD40 ligands, secrete IL-12 and efficiently stimulate T cells to proliferate and kill cancer cells [96,97,98]. Mature DCs initiate and control the amplitude of T-cell-dependent anti-tumor immunity. However, if the abovementioned processes do not occur in sequence, immune suppression mediated by T regs may be the outcome [93,94,95]. Tumors can also circumvent the abovementioned signals by loss of tumor antigens or by the release of soluble molecules and make DC function less efficient [99,100].

The inhibitory environment in the TME with TGF-β, IL-10, ARG-1, VEGF, and other molecules fosters the development of CD14+CD1a+ DCs (immature DCs), TAMs, and Tregs, which are associated with immune tolerance, immune exhaustion, and tumor growth. Preclinical studies showed that deletion of immature DCs (lacking co-stimulatory molecule CD80 and expressing low CD86) in mice bearing established ovarian tumors delayed tumor growth [101]. In translational medicine, the concept of harnessing anti-tumor DCs for improved HGSOC outcome has been recently described in clinical trial NCT00703105 [102] and in other vaccine trials using various DC modifications [7,103,104].

There are several DC types and classifications [93]. Here, in the context of cancer, it is noteworthy to mention the plasmacytoid DC (pDC; CD4+CD123+BDCA2+). These cells express CCR4 and migrate into the ovarian tumor drawn by CXCL12 (stromal-derived factor; SDF-1) and IL-10 [105]. These tolerogenic DCs release IDO, an enzyme that catalyzes tryptophan degradation [106,107]. IDO is associated with tumor angiogenesis, cancer metastasis, and immune-suppressive functions of TILs [108]. In a cohort of 44 ovarian cancer patients, pDCs in tumors were associated with early relapse [109]. In another study, higher pDC scores were independently associated with shorter PF/S [110].

### 2.6. Importance of Natural Killer Cells in Ovarian Cancer

Natural killer (NK) cells are known for their anti-cancer properties. However, ovarian cancer patients exhibit a reduction in NK cell numbers and limited function in the hostile TME and in peripheral blood [111,112,113,114,115]. The main surface antigens on NK cells are CD56, CD16, NKG2D, NKp30, NK44, and NK46, which help them to detect infected or tumor cells. CD56 bright NK cells are exceptional at producing cytokines, whereas CD56 dim NK cells have potent cytotoxic functions [114,115,116,117]. NK cells in ascites are enriched for CD56 bright cells, mostly exhibiting a cytokine secretory function rather than the CD56 low-cytotoxic NK cell subset [118,119].

NKp30, NK44, and NK46 are cytotoxicity receptors that trigger direct target killing [114]. CD16 (FcγRIIIa) plays an important role in antibody-dependent cellular cytotoxicity (ADCC) by binding IgG-coated targets [120]. In addition, NK cell activity is controlled by a balance of inhibitory or activating signals from lineage-encoded receptors, including two categories of MHC class-1-like binding receptors [111]. These receptors include a non-polymorphic C-type lectin-like receptor, such as killer cell-like receptor, K1(KLRK1), also called natural killer group 2 member D (NKG2D), which binds to stress-induced ligand, NKG2DL, on abnormal cells [121]. The NKG2DL consists of six members, MICA, and MICB, and six ULBP members, which induce activating signals leading to cytotoxicity [122]. Inhibitory receptors such as CD94/NKG2A mainly bind non-classical MHC-1b molecules such as human leukocyte antigen (HLA-E) [111,123], and inhibitory signals are released. These interactions are important to suppress NK cell activity during cytolysis. NK cells also express killer cell inhibitory receptors (KIRs), which recognize MHC class-1a molecules (HLA-A, HLA-B, and HLA-C). KIR and MHC-1 molecules binding on the surface of normal cells maintain immune tolerance in vivo and release inhibitory signals so that NK cells do not attack normal cells [111,124].

NK cells in the ovarian TME exhibit pro-cancer activities through several mechanisms [125]. For example, tumor-associated T and NK cells in ascites showed defective signaling proteins, including reduced TCR-zeta chain and p56 lck expression, as well as low levels of IFN-γ, IL-2, and IL-4 gene expression [126,127]. Ovarian cancer cells can successfully evade detection by NK cells by proteolytic shedding of molecules such as MICA and MICB (stress-induced ligands that activate the NKG2D receptor) from their surface. With this shedding activity, MICA/B can downregulate NKG2D on NK cells and lessen the anti-cancer activity of NK cells [122,128,129].

Efficient functioning NK cells have multiple anti-tumor mechanisms in cancer, including through the perforin (punches holes in tumor cell membranes)/granzyme pathway (lyse cancer cells). In ADCC, the Fc portions of their receptors bind to IgG antibodies attached to antigens on tumor cells, leading to granule release and cell lysis [116,120,130]. Cytokines such as IFN-γ and TNF-α elaborated from NK cells can limit tumor cell growth and sensitize tumor cells to conventional cancer treatments. Engineered NK cells can lyse ovarian cancer stem-like cells [113]. A rapidly growing field for clinical trials aimed at restoring NK cell function is chimeric antigen receptor (CAR) NK therapy, in which NK cells are genetically engineered with chimeric antigen receptors to recognize specific tumor antigens [114,131]. NK cell immunotherapy may have potential in ovarian and other hard-to-treat solid cancers.

### 2.7. Innate Lymphoid Cells

Innate lymphoid cells (ILCs) are a significant part of the innate immune system. The innate lymphoid cell (ILC) family is composed of NK cells, ILC1, ILC2, and ILC3 cells [132,133]. These three groups of ILCs were also revealed by sc-RNA-seq in a study profiling NK cells and ILCs in HGSOC ascites and healthy-donor peripheral blood [118]. ILC1 cells produce TNF-α and IFN-γ and share functional and transcriptional properties with NK cells [134]. ILC2 cells secrete IL-4, IL-5, and IL-15, and are associated with ovarian cancer progression [135]. There was an increased frequency of ILC2 cells with higher PD-1 expression levels in ascites from patients with HGSOC with shorter survival [118]. ILC3 cells produce IL-17 and IL-22 and contribute to mucosal immunity [133,136].

### 2.8. TANs in the TME

In 2009, investigators proposed that tumor-associated neutrophils (TANs) like TAMs could be categorized into N1 and N2 cells [137]. N1 TANs possess anti-tumor properties such as the release of immunostimulatory cytokines and chemokines and increased cytotoxicity against tumor cells [138]. N1 TANs recruit CD8+ T cells by releasing T-cell-attracting cytokines, such as CXCL9 and CXCL10, and Th1 cytokines, such as IL-12 [139]. N2 TANs secrete molecules including VEGF, ECM degrading enzymes (MMPs), IL-10, and TGF-β (Table 1), which aid in remodeling the TME and promoting tumor cell invasion and metastasis [140].

An interesting feature of neutrophils that play an important role in malignancy is neutrophil extracellular trap (NET) formation [141,142]. NETs were originally described as a mechanism whereby neutrophils trap and destroy micro-organisms but are now also known to be critical mediators of cancer-associated inflammation. NETs released by neutrophils in response to pathogens consist of fibrous decondensed chromatin bound with histones, myeloperoxidase (MPO), and cytoplasmic proteins such as elastase, cathepsin, and lactoferrin [64,143].

In tumors such as ovarian cancer, neutrophils infiltrate the omentum in early-stage ovarian cancer. Neutrophils differentiate into TANs in the presence of tumor-derived soluble molecules and hypoxia [144,145]. NET formation occurs via suicidal or NETosis pathways. Both of these pathways depend on the coordinated activity of neutrophil elastase (NE) MPO and protein–arginine deiminase 4 (PAD4) to drive chromatin decondensation and extracellular release of DNA fibers [144,145,146,147].

**Table 1:** Main factors elaborated by immune cells in the TME.

Cell Groups ^a^	Main Features	Predominant Molecules Secreted
**Anti-tumor polarization**		
CD8+ cytotoxic T cells, CD4+ helper cells, CD3+ T cells	Elicits anti-tumor immune responses. Kills tumor cells. Prolongs PF/S and O/S	IFN-γ, TNF-α, IL-2, CXCL9, CXCL10, CXCL11, CCL3, CCL4, CCL5, perforin, GrB [148,149,150]
M1 TAMs	Enhances anti-tumor responses	IFN-γ, TNF-α, IL-12, IL-18, IL-23 [151,152,153]
Mature DCs	Antigen presentation. Boosts anti-tumor responses.	IL-12, TNF-α, CXCL9, CXCL10 [148,150,154,155]
N1 TANs	Immune stimulation. Recruits CD8+ T cells	TNF-α, CXCL9, CXCL10, IL-12, CCL3 [156,157]
NK cells	Efficient tumor cell killing (ADCC and granzyme B)	TNF-α, IFN-γ, IL-2, IL-15, IL-18, CXCL9, CXCL10, CXCL11, CCL3, CCL4, CCL5, perforin, GrB [150,158]
**Pro-tumor polarization**		
T regs, PD-1+, CTLA-4+, TIM-3+, LAG-3+ or TIGIT+ expressing	Downregulation of immune responses. Immune exhaustion	TGF-β, IL-10 [159,160,161]
M2 TAMs	Invasion, metastasis, tumor cell migration and EMT	IL-10, TGF-β, CCL2, VEGF, EGF, HGF, CCL18 [66,150,152,153]
MDSCs	Reduction in immune responses, angiogenesis, invasion, therapy resistance	IL-10, TGF-β, CCL2, CCL18, CXCL1, COX-2, PGE-2, IDO, ARG-1, NOS-2, FGF, VEGF, MMPs [87,88,162]
Immature DCs	Reduced antigen presentation. Enhanced tumor escape	IL-10, TGF-β [163,164]
Plasmacytoid DCs	Downregulation of immune responses, recruits T regs	IDO [165]
N2 TANs	Pre-metastatic niche formation, invasion, metastasis, chemotherapy resistance	IL-10, TGF-β, CXCL13, VEGF, MMPs, MPO, PAD4 [156,166,167,168]
Tumor NK cells	Inefficient cell killing. Reduction in immune responses	IL-4, IL-5, IL-10, IL-13 [150,158]

^a^ Anti-tumor polarized cells elaborate soluble molecules, which enhance immune responses and kill tumor cells. Cells with pro-tumor polarization secrete molecules associated with T-cell exhaustion, downregulation of immune responses, tumor progression, and metastasis.

In the ovarian TME, NET formation is fostered by high levels of cytokines such as IL-8, complement factors, and pathogen-associated molecular patterns (PAMPs) [169]. NETs can contribute to the recruitment of IL-10 secreting B cells through NET-induced CXCL13 expression. This can lead to an increase in T regs through the secretion of IL-10, a process which contributes to immune suppression limiting T-cell proliferation [144]. Additionally, ascites and the omental architecture provide an environment where MDSCs and TANs sustain NET production through CXCR1 and CXCR2 signaling. These NETs provide an adequate system for tumor cell adhesion, dissemination of tumor cells, ECM degradation, angiogenesis, and tumor cell evasion [170]. In HGSOC, NETs are also involved with chemotherapy resistance and suppression of immune responses, and are therefore adequate culprits to concurrently aid in disease progression and to decrease the efficacy of conventional and other therapy regimens [142,170]. Thus, through a very complex and multifactorial program, as previously described by others, NETs may act as key mediators of ovarian cancer progression. Targeting of NETs is therefore potentially a novel and relevant form of therapeutic intervention. A more detailed summary of factors elaborated by immune cells in the TME is shown in Table 1.

## 3. Other Players in the Ovarian Cancer TME

In addition to the immune cells detailed above, the ovarian TME is regulated by the ECM, fibroblasts, endothelial cells, blood vessels, mesothelial cells, adipocytes, and other components. The text below covers examples of how these lesser-mentioned elements modulate immune suppression in the TME [171].

### 3.1. Extracellular Matrix Involvement in Ovarian Cancer

The mesothelium, the squamous epithelium covering organs of the peritoneal cavity has a single layer of mesothelial cells, and below this a basement membrane. The ECM consists of collagen fibers that provide strength, proteoglycans that provide cell shelter, and glycoproteins such as laminin and fibronectin that connect collagen and proteoglycans such as integrins and hyaluronic acid receptors [172,173]. The ECM provides biochemical and biomechanical stimuli that alter the function of cells in the tumor by activation of receptors including integrins and syndecans [174]. Tumor cells have abnormal ECM deposition and this may increase mechanical stiffness of these cells leading to proliferation, invasion, and cell survival [175]. The ECM is negatively regulated by immunosuppressive soluble molecules (e.g., TGF-β) released by other cell types such as TAMs, M2 cells, and T regs [176,177]. In ovarian cancer, ECM remodeling facilitates the invasion of cancer cells into surrounding tissue and metastasis to various sites in patients [178].

### 3.2. Cancer-Associated Fibroblasts

Fibroblasts in the stroma regulate the pre-metastatic niche of ovarian cancer by remodeling the ECM and releasing cytokines. Under the influence of proliferative/activation signals, such as TGF-β, these fibroblasts differentiate into activated fibroblasts, known as cancer-associated fibroblasts (CAFs) [179,180]. CAFs are the main component of ovarian cancer stromal cells and are located in close proximity to ovarian cancer cells [181,182,183]. CAFs are spindle-shaped cells that are known for their response to tissue injury and secretion of several soluble molecules for endothelial cell recruitment during angiogenesis, including TGF-β, platelet-derived growth factor (PDGF), and fibroblast growth factor-2 (FGF-2). They release several cytokines that stimulate proliferation, invasion, and metastasis of tumor cells in their vicinity and indirectly regulate immune cells [179,184,185].

CAFs promote tumorigenesis by releasing high levels of mitogenic factors such as fibroblast growth factor-1 (FGF-1) and HGF [186,187]. Others showed that IL-8 secreted by CAFs and ovarian cancer cells promotes stemness through the activation of Notch-3-mediated signaling pathways [188]. CAF-secreted CXCL14 drives the expression of long noncoding RNA LINC00092 in ovarian cancer cells and promotes ovarian cancer growth and invasion [189].

A group of investigators subdivided HGSOC into four molecular subtypes. The mesenchymal HGSOC subtype was defined by stromal-related gene signatures and was associated with poor patient survival. These workers also identified four subsets of CAFs (CAF-S1-4) based on CD29, fibroblast activation protein (FAP), fibroblast-specific protein-1 (FSP-1), and smooth muscle actin (SMA) protein levels. CAF-S2 and CAF-S3 were classed as “non-activated” CAFs based on the lack of expression of SMA. CAF-S1 and CAF-S4 both expressed SMA and were classified as activated CAFs or myofibroblasts. CAF-S1 cells were one of the most frequent CAF subtypes in mesenchymal HGSOC. CXCL12α was expressed at similar levels in both CAF-S1 and CAF-S4 subsets. However, the beta isoform of chemokine CXCL12 (CXCL12β) specifically accumulated in the CAF-S1 cell subset but was almost undetectable in CAF-S4 cells. These CAF-S1 cells were highly immunosuppressive and enhanced the attraction, survival, differentiation and suppressive activity of CD25+FoxP3+ T cells. CXCL12β expression in CAF-S1 cells was crucial to the immune-suppressive capacity of these cells [190]. The identification of CAF subtypes crucial to ovarian cancer outcome will allow for more precise targeting of these cells in therapy design.

CAFs can exert compressive forces on micro-vessels and form physical barriers, which may make the delivery of chemotherapeutic agents less effective and lead to chemoresistance [191]. CAFs activate the Wnt/β-catenin pathway in ovarian cancer via the CXCL12/CCR4 linkage, thereby inducing EMT and cisplatin resistance [192]. CAFs, through matrix metalloproteinase (MMP) production, stimulate the degradation of the vascular basement membrane (a layer of the ECM). CAFs contribute to increased deposition of and alteration in the ECM and this creates a barrier that blocks efficient delivery of anti-cancer drugs and enhances chemoresistance. It is also known that fibroblasts diminish nuclear accumulation of platinum in ovarian cancer cells, enhancing resistance to platinum-based chemotherapy [183,187,193]. This resistance is partly due to glutathione and cysteine released by fibroblasts. However, CD8+ T cells can alter cystine and glutathione metabolism in CAFs via the JAK/STAT pathway, and this reduces CAF-induced resistance to platinum therapy [193].

### 3.3. Cancer-Associated Endothelial Cells and Angiogenesis

Endothelial cells line the inside of blood vessels. In cancer, these are the blood vessel-lining cells in the TME that supply nutrients and oxygen to the rapidly proliferating tumor cells [194]. Angiogenesis is the forming of new capillaries from existing blood vessels, a process that leads to the continued growth and survival of tumor cells by various mechanisms [194,195,196]. For example, CAFs through MMP production stimulate the degradation of the vascular basement membrane, leading to endothelial cell proliferation and migration. Pro-angiogenic factors such as vascular endothelial growth factor-A (VEGF-A) are normally in equilibrium with anti-angiogenic factors such as thrombospondin, and this maintains a state of tumor quiescence [196]. Under hypoxic conditions, hypoxia-inducible factors (HIFs) become active. With tumor hypoxia, HIF-1α is stabilized, leading to increased expression of pro-angiogenic factors including VEGF, PDGF, and FGF-2, and a definite suppression of anti-angiogenic factors [196]. HIF-1-induced VEGF upregulation along with MMP-9 increase leads to higher concentrations of soluble VEGF, followed by increased angiogenesis and metastasis [197]. The switch from an anti-angiogenic to a pro-angiogenic state creates an immune-suppressive TME and increases the abundance of pro-tumor TILs [195,198].

The importance of VEGF family members in action in the angiogenic process is presented in the subsequent text. VEGF binds to its receptors VEGFR-1 and VEGFR-2 on endothelial cells and trigger intracellular signals that cause endothelial cells to divide and increase vessel mass and produce new branches of vessels. It also prevents the apoptosis of endothelial cells and makes blood vessels more permeable to gases and nutrients that nourish the tumor cells [199]. VEGF-C in patient ascites is associated with FIGO stage, tumor grade, and lymph node metastasis, and a high concentration of VEGF is a predictor of reduced survival [200]. The angiogenesis inhibitor bevacizumab is a useful therapeutic option in ovarian cancer and acts by blocking VEGF-A and inhibiting blood vessel growth [201].

### 3.4. Cancer-Associated Mesothelial Cells

Mesothelial cells are supposed to be a defense barrier against ovarian cancer metastasis into the abdominal cavity but may also act adversely to establish niches of tumor cells that promote ovarian cancer metastasis [202,203,204,205]. CAMs secrete chemokines that promote peritoneal metastasis and chemoresistance in ovarian cancer. They also secrete high levels of IL-6 and FGF, which promote ovarian cancer progression [206,207]. Lipophosphatidic acid (LPA) secreted by CAMs enhanced adhesion, migration, and invasion of ovarian cancer cells [208,209].

Investigators performed sc-RNA analysis of cancer stroma-derived cells using 18,403 cells unbiasedly collected from seven untreated HGSOC tumors [210]. This allowed the identification of transcriptional markers specific to each stromal phenotype. From these markers, they estimated the prevalence of these phenotypes in conventional bulk data sets and characterized the relevance of these stromal phenotypes to patient prognosis. These workers determined six cellular phenotypes linked to prognosis as follows: mesothelial cells (FB_CALB2), myofibroblasts (FB_MYH11), TGF-β-driven cancer-associated fibroblasts (FB_COMP), tumor subcluster Tum_BAMBI, and lymphatic endothelial cells (EC_PROX1), which were associated with adverse effects on outcomes. Only the plasma cell subcluster (BC_IGHG1_PRDM1high) was associated with improved OS [210].

### 3.5. Cancer-Associated Adipocytes

The omentum, a prevalent site of ovarian cancer metastasis and recurrence, is rich in adipose tissue [211]. Under normal conditions, adipocytes are specialized for lipid storage and endocrine signaling. In cancer, these cells are dysregulated in function due to the immunosuppressive environment in which they exist [211]. Cancer-associated adipocytes (CAAs) induce ovarian cancer homing by secreting adipokines, leptin, adiponectin, IL-6, IL-8, MMP-11, and CCL5 [211]. Adipocyte-derived signals such as inflammatory mediators can enhance lipid uptake in ovarian cancer via STAT3/fatty acid-binding protein 4 (FABP4) signaling, leading to an increased proliferation of cancer cells [212]. An adipose-rich microenvironment fosters ovarian cancer growth, cell proliferation, and migration, as well as chemoresistance through several mechanisms [211,213,214].

## 4. Novel Treatment Strategies for Ovarian Cancer

The previous discussion underscores the diverse and intricate factors that confer potent immune suppression in the ovarian TME. It is therefore likely that therapeutic efficacy in HGSOC will be optimized if treatment agents are used together to better overcome the many barriers to anti-tumor immunity, which exists in the TME. Some of the recent advances in ovarian cancer therapies are discussed in the subsequent text.

### 4.1. Immune Checkpoint Inhibitors

For the greater part, immune checkpoint inhibitors have revolutionized cancer treatment [37,38,39]. Cancer cells circumvent immune surveillance through the activation of IC pathways, thereby inhibiting T-cell activation and limiting anti-tumor responses. Co-inhibitory molecules on the surface of tumor cells or antigen-presenting cells bind to T-cell co-inhibitory molecules and immune checkpoints [35]. The FDA approved the first of these ICI agents, Ipilimumab, in 2011, to target CTLA-4 in metastatic melanoma. Since then, other ICIs have been FDA-approved to target CTLA-4 as well as the PD-1/PD-L1 axis for a range of cancers such as renal cell carcinoma, colorectal cancer, hepatocellular carcinoma, non-small-cell lung cancer, and Hodgkin lymphoma amongst others [37,38,39,40].

Generally, ICIs have only yielded limited success in ovarian cancer [41,42,43,44,215,216]. However, notable progress in this area has been recently achieved. In 2026, following the evaluation of the KEYNOTE-B96 (NCT05116189) clinical trial (643 patients enrolled) [13], the FDA approved pembrolizumab (Keytruda) as well as pembrolizumab and berahyaluronidase alfa-pmph (Keytruda Qlex) in combination with paclitaxel, with or without bevacizumab, for adults with platinum-resistant epithelial ovarian, fallopian tube, or primary peritoneal carcinoma with tumors expressing PD-L1 (combined positive score; CPS ≥ 1), as determined by an FDA-approved test, and who have received one or two prior systemic therapy regimens.

There are several other clinical trials for ovarian cancer that are ICI-based [217,218], including NCT03602859 and NCT03737643, both testing anti-PD-1 and PARP inhibitors (PARPis), to mention a few. Additionally, a phase I clinical trial, NCT04503980, evaluates anti-PD-1 and mesothelin (MSLN)-directed CAR T cells.

### 4.2. Targeting TAMs in Ovarian Cancer

Therapies targeting M2 TAMs may be designed to shift them to M1 immunocompetent cell polarization. Potential agents to use are antibodies or inhibitors against colony stimulating factor-1 (CSF-1) receptor, against CCL-2 or against CD11b [219,220,221]. Triggering receptor expressed on myeloid cells (TREM)-1 found on MDSCs promotes tumor growth and suppresses anti-tumor T-cell responses predisposing T cells to T-cell exhaustion [222,223]. Effective inhibitors of TREM-1 may be designed for future clinical trials. Overall, testing of agents blocking TAM activity in cancer is predominantly in the pre-clinical phase [64,224].

### 4.3. Dendritic Cell-Based Immunotherapies for Ovarian Cancer

The use of DC vaccine therapy holds much promise in cancer treatment [225]. Typically, this involves the propagation of autologous myeloid-derived DCs from blood monocytes, pulsing them with tumor antigens and maturing them with cytokines, before infusion into patients for the generation of patient tumor-specific T-cell responses.

DC therapy has shown some efficacy in advanced ovarian cancer using several treatment regimens. For example, investigators administered a mature α-DC-1 vaccine (NCT00703105) to patients with relapsed/refractory ovarian cancer with minimal residual disease (MRD) after secondary debulking surgery. In the small cohort of 19 patients enrolled in the trial, this vaccine was found to be safe and in 26% of the patient cohort OS exceeded five years. Furthermore, administration of repeated vaccine administrations (six or more vaccines) was associated with a significant improvement in PFS [102]. Even though several other DC vaccines have been reported to show safety and some level efficacy in HGSOC [7,103,104], there is need for improvement in patient outcomes for DC vaccine therapy in all cancers tested. The immunogenicity of DC vaccines may be enhanced by combining this therapy with T-cell targeting therapies such as ICI or other agents.

Lastly, in the area of pDC targeting, in a phase I clinical trial, an IDO inhibitor, Epacadostat, was used in ovarian cancer patients (NCT02042430).

### 4.4. Inhibition of Angiogenesis

Bevacizumab (FDA-approved in 2014) is an anti-VEGF-A antibody, which inhibits angiogenesis and prevents the tumor from growing [226,227,228]. Apart from conventional treatment for HGSOC, bevacizumab in combination with platinum-based chemotherapy, followed by maintenance bevacizumab, became the first targeted therapy option for newly diagnosed HGSOC patients, based on the outcome of the GOG-0218 study [229,230,231,232,233].

Bevacizumab also shows efficacy when combined with, and as maintenance following, platinum-based chemotherapy in platinum-sensitive, recurrent ovarian cancer patients, and in combination with paclitaxel, pegylated liposomal doxorubicin, or topotecan in platinum-resistant, recurrent ovarian cancer patients [227,234]. After the PAOLA-1 trial analysis [235,236], bevacizumab combined with the PARP inhibitor (PARPi) olaparib was approved as maintenance therapy in patients with newly diagnosed advanced ovarian cancer with homologous recombination deficiency (HRD)-positive tumors (known as a *BRCA1* and/or *BRCA2* mutation and/or genomic instability) who achieved a clinical response after platinum-based chemotherapy plus bevacizumab [227]. Another ovarian cancer clinical trial designed to target angiogenesis includes phase III NCT00532194 targeting VEGFR1-3.

### 4.5. PARP Inhibitors

PARPis include agents such as olaparib, nivaparib, rucaparib, and others, which block the repair of single-stranded DNA breaks, leading to replication-associated double-stranded breaks (DSBs). In HRD ovarian cancer cells, these DSBs are not efficiently repaired, leading to cell death. PARPis have shown efficacy especially in patients with BRCA mutations or in HRD patients [9,237,238]. Clinical trials for PARPis, which show efficacy in ovarian cancer include phase III trials such as SOLO1 (evaluating olaparib as maintenance therapy in newly diagnosed patients), SOLO2 (evaluating olaparib for platinum-sensitive recurrent patients), and PRIMA (testing niraparib as a first-line maintenance therapy in patients with advanced ovarian cancer) [239,240,241,242,243]. Based on the outcome of the SOLO2 trial and Study 19 [244], olaparib received full approval as maintenance therapy in 2017 following the response to platinum-based chemotherapy. There are several other PARPi clinical trials that provide much-needed therapy options for ovarian cancer patients [9,237].

### 4.6. Targeting the TME with Antibody Drug Conjugates

Antibody drug conjugates (ADCs) deliver cytotoxic payloads using target antibodies. Important targets in ovarian cancer are folate receptor alpha (FR-α), human epidermal growth factor-2 (HER-2), MSLN, and cadherin-6 (CDH6) [245,246]. Miretuximab soravtansine (MIRV), an ADC that targets FR-α on tumor cells, received accelerated FDA approval (2022) for FR-α high, platinum-resistant ovarian cancer following 1–3 lines of therapy based on the outcome of the SORAYA trial (NCT04296890) [247]. This trial reported a confirmed objective response rate (ORR) of 32.4% in a biomarker-selected population. The confirming MIRASOL trial (NCT04209855) met its primary endpoint of PFS [12]. The phase III GLORIOSA trial (NCT05445778) evaluates MIRV plus bevacizumab as single maintenance therapy in FR-α high platinum-sensitive ovarian cancer.

### 4.7. Miscellaneous TME Targets

A phase III trial evaluated O/S with relacorilant and nab-paclitaxel in patients with platinum-resistant ovarian cancer (NCT05257408, ROSELLA) [248]. Relacorilant is a selective glucocorticoid receptor antagonist that increases the sensitivity to chemotherapy in several cancer types. This study’s cohort consisted of patients who had one-to-three lines of previous anti-cancer therapy and platinum-resistant disease. The use of relacorilant with nab-paclitaxel resulted in significantly longer O/S in patients with platinum-resistant ovarian cancer, without the need for biomarker selection [248].

A bispecific antibody, NI-1801 (targeting mesothelin and CD47), has also recently been evaluated with promising success. Study LCB-1801-001 is an open-label, phase 1 first-in-human clinical study of NI-1801 in patients with advanced, metastatic, or recurrent solid malignancies (including ovarian cancer) expressing mesothelin (MSLN). Unpublished meeting reports currently indicate that this agent in combination with pembrolizumab resulted in 40.2 percent of heavily pretreated ovarian cancer patients being alive at two years (NI-1801 Mesothelin x CD47 Bispecific Two-Year Ovarian Data), thereby indicating the potential success of strategies targeting mesothelial cells and ICs in the TME in this disease.

BCA101 is a bi-functional fusion antibody that simultaneously blocks EGFR and TGF-β signaling pathways in the TME to suppress tumor growth and enhance anti-tumor immune responses. Based on the findings of pre-clinical studies, clinical trials targeting CAFs include NCT04429542 using BCA101 [249] as single therapy or with ICI pembrolizumab in ovarian and other solid cancer patients. A phase 1 clinical trial (NCT03078400) evaluated the CD44 blocking peptide (targeting the ECM) plus chemotherapy. Evaluation of anti-MSLN (targeting CAMs), VEGFRi, and chemotherapy is in a phase I clinical trial (NCT04938583) for ovarian cancer patients.

### 4.8. Enhancement in T- and NK Cell Function in Ovarian Cancer

There are many types of adoptive T-cell therapies under development and being tested for ovarian cancer [250]. CAR T cells are MHC-unrestricted, directly binding to target antigens without the need for antigen presentation by peptide–MHC complexes. CARs are composed of an extracellular antigen-binding domain fused with the signaling and co-stimulatory domains of TCR. The extracellular domain is composed of a single-chain variable fragment (scFv), a fusion protein of the variable regions of immunoglobulin heavy and light chains, for the specificity of the treatment. The intracellular domains contain the primary CD3 signaling domain and secondary costimulatory domains, and some constructs add inducible cytokine expression to recruit bystander T cells for improved immune-mediated anti-tumor effects and clonal amplification [10,11,251]. CAR T-cell therapy works well in hematologic malignancies [252,253]. For ovarian cancer, this strategy is in the early phase of clinical testing. Clinical trial NCT04503980 is currently investigating MSLN-CAR T cells secreting PD-1 nanobodies in ovarian cancer.

There are several strategies in pre-clinical studies and in clinical trials to restore NK cell persistence and anti-tumor activity in ovarian cancer [114,254,255]. For example, a phase 1B trial (NCT06321484) is investigating patient-derived cytokine-induced memory-like NK cells (IL-12, IL-15, IL-18) with chemotherapy. CAR NK cells are genetically engineered to directly recognize tumor antigens. CAR NK cells can utilize mismatched allogeneic cells, and the risk of cytokine release syndrome and of graft versus host disease is low [114,256]. CAR T-cell and CAR NK cell therapy clinical trials for ovarian cancer are primarily in the first phase, and much work still needs to be done for the advancement of this field [257].

Table 2 provides a summary of selected clinical trials designed to target immune suppression in the ovarian TME.

## 5. Perspectives

The preceding text outlines a complex, heterogenous, and dynamic ovarian TME in which cells can alter phenotype and function depending on the concentration of cytokines, chemokines, or other soluble molecules in their environment. Generally, a paucity of immunocompetent T cells, DCs, and macrophages are found in the TME. On the contrary, there is an abundance of pro-tumor cell types, including MDSCs, M2 TAMs, N2 TANs, and Tregs, as well as other phenomena, such as loss of tumor cell surface antigens (such as MHC-I), TP53 mutations, or tumor cells altering specific signaling pathways, which favor tumorigenicity [128,262]. Cell types with pro-tumor propensity may secrete IL-10, TGF-β, VEGF, or CCL2 and other agents, which promote the recruitment of T regs, increase angiogenesis, metastasis, and drug resistance. It is therefore evident that there are many layers of immune suppression in the ovarian TME.

The inherent complex immunosuppression in the HGSOC TME presents challenges in designing effective therapy for this disease because such treatment agents are needed to address multiple factors, such as ECM remodeling, restoring function to the tumor vasculature, reducing T regs, restoring T-cell, DC, and/or NK cell function. To this end, there are many exciting clinical trials that are designed as combinatorial studies to target some of these immunosuppressive targets together [128,262] (Table 2). Several of these trials are testing the safety and efficacy of treatments, but they will also need to test the frequency and sequence of administration of agents in combination regimens, which will lead to optimum O/S.

Despite this aggressive female malignancy, there is hope on the horizon because of the many scientific advancements that are being made concurrent with clinical efforts, even though challenges persist. Interactions between the cancer and immune system vary between patients and are not currently understood, and this limits the development of effective combination therapies. Diagnostic tools such as spatial imaging, spatial genomics, and spatial transcriptomics will aid in the analysis of tumor cell subsets as well as detailed analysis of unique gene and protein features specific to individuals [263,264,265,266]. Spatial imaging can aid in revealing diverse immune niches, CD8+ T-cell densities, DC maturation state, and in general show immune cell localization, exhausted or dysfunctional T cells, MDSCs, and ICs such as PD-L1 in the tumor, and also the association with tumor stage and overall survival (O/S). This knowledge can be used to design combination therapy, which can reprogram cold tumors into hot tumors. High-resolution genomics, transcriptomics, and proteomics tools used to study the excised tumor can reveal region-specific proteins and RNA associated with patient survival. Newly revealed disease and treatment biomarkers as a result of these tools will make it easier to create innovative and more specific effective combination therapies to target the immune-suppressed ovarian TME. Such high-resolution TME investigations will direct patient selection, thereby narrowing treatments to cohorts that will respond best in specific clinical trials, and can also be used to more precisely measure response rates to treatments.

Improvements in delivery of treatment agents, such as by nanoparticle delivery of drugs or oncolytic viral approaches, may enhance drug stability and provide greater treatment efficacy [267,268]. Oncolytic viral immunotherapy uses naturally occurring or genetically modified viruses that can predominantly kill tumor cells as well as stimulate anti-tumor immune responses [269]. The viruses often used in preclinical or clinical systems are oncolytic adenovirus, human syncytial virus, and vaccinia virus [268]. These viruses replicate in tumor cells leading to tumor cell death, while normal cells are cleared of the virus. Pathogen-associated molecular patterns (PAMPs) and damage-associated molecular patterns (DAMPs), tumor antigens, and viral antigens are released, which can boost DC-T-cell priming, induce antigenicity, and lead to the recruitment of cytotoxic effector T cells, thereby reprogramming a cold tumor to a hot tumor, thus making the TME more responsive to immunotherapy, such as ICIs [11,270]. Oncolytic viral therapy is well-tolerated but, to date, the desired clinical efficacy has not been achieved in cancer therapy [271,272].

Nano-drug delivery systems may overcome some of the difficulties of specifically targeting tumor sites with treatment agents and reduce damage to normal tissues when therapy is administered. Nanoparticles and nanocapsules may be useful for the delivery of chemotherapy drugs and targeted therapies. In one case, a multi-drug loading nanosystem incorporating digoxigenin (Dig) enhanced the chemotherapeutic effect of ovarian cancer through chemotherapy and induced immunogenic cell death (ICD) [273]. It is anticipated that current and developing technological advances will lead to innovative therapies in the future, which will remodel the ovarian TME and significantly impact the O/S of HGSOC.

## Figures and Tables

**Table 2:** Highlights of clinical trials targeting the ovarian TME.

NCT Number	Agents	Study Title	Phase/Number Enrolled	Median PF/S (months)	Median OS (months)/ Outcomes
NCT05116189 ENGOT-ov65/ KEYNOTE-B96	Study arm: Pembrolizumab and Chemotherapy (CT) Control arm: Placebo and CT	A Phase 3, Randomized, Double-Blind Study of Pembrolizumab Versus Placebo in Combination With Paclitaxel With or Without Bevacizumab for the Treatment of Platinum-resistant Recurrent Ovarian Cancer (KEYNOTE-B96/ENGOT-ov65)	Phase III Completed N = 643	8.3 vs. 6.4 [13] HR 0.70, 95% CI, 0.58–0.84; *p* < 0.0001 [13]	17.7 vs. 14.0 HR 0.82, 95% CI, 0.69–0.97; *p* = 0.011 Led to FDA approval of pembrolizumab in platinum resistant disease with paclitaxel +/− bevacizumab; PD-L1 expression (CPS ≥ 1)
NCT03737643 DUO-O	CT + bevacizumab + durvalumab, maintenance bevacizumab + durvalumab. CT + bevacizumab + durvalumab, maintenance bevacizumab, durvalumab, olaparib Control: CT + bevacizumab, maintenance bevacizumab.	A Phase III Randomised, Double-Blind, Placebo-Controlled, Multicentre Study of Durvalumab in Combination With Chemotherapy and Bevacizumab, Followed by Maintenance Durvalumab, Bevacizumab and Olaparib in Newly Diagnosed Advanced Ovarian Cancer Patients (DUO-O).	Phase III Completed N = 1130	25.1 vs. 19.3 for maintenance triplet vs. control HR 0.62, 95% CI, 0.52–0.73 [258] 20.6 for CT with bevacizumab + durvalumab HR (vs. control) 0.84, 95% CI, 0.71–0.99	No OS benefit for experimental arms.
NCT00262847 GOG-0218	CT plus concurrent bevacizumab CT plus concurrent and maintenance bevacizumab Control: CT	A Phase III Trial of Carboplatin and Paclitaxel Plus Placebo Versus Carboplatin and Paclitaxel Plus Concurrent Bevacizumab (NSC # 704865) Followed by Placebo, Versus Carboplatin and Paclitaxel Plus Concurrent and Extended Bevacizumab, in Women With Newly Diagnosed, Previously Untreated, Stage III or IV Epithelial Ovarian, Primary Peritoneal or Fallopian Tube Cancer	Phase III Completed N = 1874	14.1 (bevacizumab throughout) vs. 11.2 (bevacizumab initiation) vs. 10.3 (control) [232,233] HR for progression or death 0.908, 95% CI, 0.795–1.040; *p* = 0.16 with bevacizumab initiation and 0.717, 95% CI, 0.625–0.824; *p* < 0.001 with bevacizumab throughout.	No OS benefit for the experimental arms. Served as basis for FDA approval of bevacizumab in frontline treatment with carboplatin and paclitaxel, followed by single-agent maintenance bevacizumab.
NCT02477644 PAOLA-1/ENGOT-ov25	Olaparib with maintenance bevacizumab Control: Placebo with bevacizumab	Randomized, Double-Blind, Phase III Trial Olaparib vs. Placebo Patients With Advanced FIGO Stage IIIB-IV High Grade Serious or Endometrioid Ovarian, Fallopian Tube, or Peritoneal Cancer Treated Standard First-Line Treatment	Phase III Completed N = 806	22.1 vs. 16.6 [235,236] HR for disease progression or death, 0.59, 95% CI, 0.49–0.72; *p* < 0.001.	56.5 vs. 51.6 HR 0.92, 95% CI, 0.76–1.12; *p* = 0.4118. Led to FDA approval of olaparib with bevacizumab as first-line maintenance for advanced HRD-positive ovarian cancer.
NCT01844986 SOLO1/GOG 3004	Olaparib Control: Placebo	A Phase III, Randomised, Double Blind, Placebo Controlled, Multicenter Study of Olaparib Maintenance Monotherapy in Patients With BRCA Mutated Advanced (FIGO Stage III-IV) Ovarian Cancer Following First Line Platinum Based Chemotherapy.	Phase III Active, not recruiting N = 391	56.0 vs. 13.8 [239,240,241] HR 0.33, 95% CI, 0.25–0.43.	HR for OS 0.55, 95% CI, 0.40–0.76; *p* = 0.0004 [*p* < 0.0001 required to declare statistical significance]. Resulted in FDA approval of olaparib maintenance therapy in BRCAm advanced ovarian cancer.
NCT01874353 SOLO2/ENGOT-Ov21	Olaparib Control: Placebo	Phase III Randomised, Double Blind, Placebo Controlled Study of Olaparib Maintenance Monotherapy in Platinum Sensitive Relapsed BRCA Mutated Ovarian Cancer Patients With a Complete or Partial Response Following Platinum Based Chemotherapy	Phase III Active, not recruiting N = 295	19.1 vs. 5.5 [242,243] HR 0.30, 95% CI, 0.22–0.41; *p* < 0·0001.	51.7 vs. 38.8 HR 0.74, 95% CI, 0.54–1.00; *p* = 0.054.
NCT01847274 ENGOT-OV16/NOVA	Niraparib Control: Placebo	A Phase 3 Randomized Double-blind Trial of Maintenance With Niraparib Versus Placebo in Patients With Platinum Sensitive Ovarian Cancer.	Phase III Completed N = 553 gBRCA cohort = 230 non-gBRCA cohort = 350	21.0 vs. 5.5 in gBRCA cohort [259,260] HR 0.27, 95% CI, 0.17–0.41; *p* < 0.001 9.3 vs. 3.9 in non-gBRCA cohort HR 0.45, 95% CI, 0.34–0.61; *p* < 0.001	No OS benefit for experimental arm. Led to FDA approval of niraparib in maintenance setting for recurrent disease due to strong PFS benefit, later restricted to BRCAm tumors.
NCT01968213 ARIEL 3	Rucaparib Control: Placebo	A Study of Rucaparib as Switch Maintenance Following Platinum-Based Chemotherapy in Patients With Platinum-Sensitive, High-Grade Serous or Endometrioid Epithelial Ovarian, Primary Peritoneal or Fallopian Tube Cancer (ARIEL3)	Phase III Completed N = 564	BRCA-mutant carcinoma: 16.6 vs. 5.4 [261] HR 0.23, 95% CI, 0.16–0.34; *p* < 0·0001 Intention-to-treat population: 10.8 vs. 5.4 HR 0.36, 95% CI, 0.30–0.45; *p* < 00001	No OS benefit for experimental arm. Led to FDA approval for maintenance in recurrent, platinum sensitive disease, then restricted to BRCAm disease.
NCT04296890 SORAYA	Mirvetuximab Soravtansine	SORAYA: A Phase 3, Single Arm Study of Mirvetuximab Soravtansine in Platinum-Resistant, Advanced High-Grade Epithelial Ovarian, Primary Peritoneal, or Fallopian Tube Cancers With High Folate Receptor-Alpha Expression	Phase II Completed N = 205	4.3, 95% CI, 3.7–5.2 [247]	15.0, 95% CI, 11.5–18.7 Met the primary endpoint with objective response rate of 32.4% (95% CI, 23.6–42.2).
NCT04209855 MIRASOL	Mirvetuximab Soravtansine	MIRASOL: A Randomized, Open-label, Phase 3 Study of Mirvetuximab Soravtansine vs. Investigator’s Choice of Chemotherapy in Platinum-Resistant, Advanced High-Grade Epithelial Ovarian, Primary Peritoneal, or Fallopian Tube Cancers With High Folate Receptor-Alpha Expression	Phase III Completed N = 453	5.62 vs. 3.98 HR 0.65, 95% CI, 0.52–0.81; *p* < 0.0001 [12]	16.46 vs. 12.75 HR 0.67, 95% CI, 0.50 to 0.89; *p* = 0.0046). Basis of FDA approval for treatment of FRα positive, platinum-resistant epithelial ovarian, after prior therapies.
NCT02107937 SOV01	Autologous dendritic cell immunotherapy (DCVAC/OvCa)	A Randomized, Open-label, Three-arm, Multi-center Phase II Trial of Addition of DCVAC/OvCa to First Line Standard Chemotherapy in Women With Newly Diagnosed Epithelial Ovarian Carcinoma	Phase II Completed N = 99	20.3 (DCVAC parallel to CT), not reached (sequential to CT), and 21.4 months (CT alone) [103] HR 0.98, 95% CI, 0.48–2.00; *p* = 0.9483 for parallel to CT vs. CT HR 0.39, 95% CI, 0.16–0.96; *p* = 0.0336 for sequential to CT vs. CT	OS data has not been reported. Addition of DCVAC/OvCa vaccine to CT significantly improved PFS in newly diagnosed epithelial ovarian cancer, with the greatest clinical benefit in immunologically cold tumors.
NCT00703105	Autologous mature DC vaccine (alpha-DC-1)	Defining the Role of CD4+CD25+ Immunoregulatory T-cells in the Treatment of Patients With Advanced Ovarian Cancer Who Receive Dendritic Cell Based Vaccine Therapies	Phase II Completed N = 19	9.7, 95% CI, 5-NA [102]	42.2, 95% CI, 31.2–68.3 5-year OS: 26.3% (5/19) ≥6 vaccine doses was significantly correlated with improved PFS.

## Data Availability

No new data were created or analyzed in this study. Data sharing is not applicable to this article.
